# Self-reported depression and its risk factors among hypertensive patients, Morocco: a cross-sectional study

**DOI:** 10.1038/s41598-024-61390-y

**Published:** 2024-05-16

**Authors:** Fatima Zahra Boukhari, Safae Belayachi, Firdaous Essayagh, Othmane Terkiba, Ahmed Anouar Naji, Mohammed Amine, Abdellah Lambaki, Meriem Essayagh, Sanah Essayagh, Touria Essayagh

**Affiliations:** 1 Faculté des sciences et techniques, Laboratoire agroalimentaire et santé, Hassan First University of Settat, Settat, Morocco; 2 Faculté des sciences juridiques, économiques et sociales, Laboratoire droit privé et enjeux de développement, Université Sidi Mohamed Ben Abdellah, Fès, Morocco; 3 Institut supérieur des sciences de la santé, Laboratoire sciences et technologies de la santé, Hassan First University of Settat, Settat, Morocco; 4 Faculty of medicine and pharmacy, Clinical research service, Mohammed VI university hospital center, Department of public health, epidemiology and community medicine, Laboratory of biosciences and health, Cadi Ayyad University, Marrakech, Morocco; 5 Faculté des Sciences de la Santé, Université de Lomé, Lomé, Togo; 6 Office national de sécurité sanitaire des produits alimentaires, Oriental, Morocco

**Keywords:** Cross sectional study, Comparative study, Depression self-reported, Hypertension, Risk factors, Risk factors, Epidemiology

## Abstract

Hypertensive patients are at an elevated risk of developing mental diseases such as depression, which can impair their quality of life. The purpose of this study is to measure the prevalence of self-reported depression among hypertensive patients treated at primary health care facilities in Marrakech. Between May 2021 and December 2022, a cross-sectional study of 1053 hypertensive patients attending primary health care facilities in Marrakech was conducted. A face-to-face questionnaire was used to collect socio-demographic, behavioral, and clinical data, as well as hypertension treatment characteristics and the care-patient-physician triad. The Patient Health Questionnaire-9 was used to assess self-reported depression. To identify self-reported depression risk factors, multivariate logistic regression was used. Depressive symptoms were reported by 56.1% of hypertensive patients. The patients' average age was 63.2 ± 9.5 years, and 508 (85.9%) were female. Female sex, stress, a low-salt diet, pain and physical discomfort, an urban living environment, a lack of self-monitoring of hypertension, an unsatisfactory relationship with the healthcare system, a family history of hypertension, and the perception of adverse effects of the antihypertensive drug were all associated with self-reported depression. Self-reported depression is prevalent among hypertensive patients in Marrakech. The mental health component should be emphasized while addressing hypertensive patients in primary health care facilities.

## Introduction

High blood pressure (HBP) is a significant public health issue. In 2019, 1.28 billion individuals worldwide were afflicted with hypertension, with 82% living in low- or middle-income countries^1^. This number is anticipated to climb by 60% by 2025, according to epidemiological studies^2,3^. Every year, HBP kills 9.4 million people. It represents a considerable financial burden for nations with 57 million disability-adjusted life years (DALYs) globally, including 658,301 DALYs in Morocco^4,5^. The prevalence of HBP was 29.3% in the most current StepWise study on risk factors for noncommunicable diseases, which was conducted on a sample of 5429 persons aged 18 and older in Morocco in 2018^5,6^. Morocco's Ministry of Health and Social Protection has been executing a countrywide hypertension prevention and control program since 1996. This initiative provides 45 million dirhams per year for the purchase of medications and equipment needed by primary health care facilities to guarantee hypertensive patient diagnosis and follow-up^7^. Primary health care facilities treated and monitored 1,250,000 hypertensive patients in 2021^7^. Despite the fact that hypertensive individuals are more likely to suffer from mental diseases such as depression^8^, we manage their physical health without addressing their mental health. Depression symptoms include sadness, loss of interest or pleasure, feelings of guilt or low self-esteem, sleep or food problems, weariness, and a lack of attention^9^. In 2017, depression was observed in 40.1% of 411 hypertensive patients in Karachi, Pakistan. In people with HBP, depression is associated with an increased risk of stroke and myocardial infarction. A delay in diagnosis can lead to problems, a decrease in the quality of life of hypertensive patients, and even death^10^.

To the best of our knowledge, no epidemiological study has looked at the mental health of Moroccan hypertensives. Given the prevalence of hypertension and its influence on mental health, research into self-reported depression in hypertensives is crucial. This would provide evidence on which policymakers in the country may depend to enhance the hypertension treatment program and meet the overall health needs of hypertensive patients. As a result, the objective of our research was to determine the prevalence of self-reported depression among hypertensive patients followed at primary health care facilities in Marrakech.

## Subjects and methods

Marrakech is a Moroccan city in the center of the country. El Kelaa des Sraghna borders it on the north, Al Haouz on the south, Rhamna on the east, and Chichaoua and Safi on the west. It has a population of 1,330,468 people and covers an area of 2625 km^2^^11^. Its primary care facilities care for and monitor 23,213 hypertensive individuals^12^.

Between 2021 and 2022, a cross-sectional study was conducted on hypertensive patients aged 18 and older who were followed at primary health care facilities in Marrakech, were on pharmaceutical therapy for HBP for at least six months, and had agreed to participate in the study. Data collection was made by convenience sampling. a two-stage stratified survey was employed. Seventy percent of hypertensives resided in cities, whereas thirty percent lived in rural regions. To select the primary unit, which is the primary health care facilities, a first draw was made from the list of primary health care facilities supplied by the representative of the Ministry of Health and Social Protection of Marrakech. A second draw was made from the patients' order numbers to determine the secondary unit, which is the hypertensive patients. These order numbers were issued to patients when they arrived at primary health care facilities in order to control the flow of hypertensives during their follow-up. Pregnant women were not permitted to take part in the study.

### Sample size

We determined our sample size based on a 50% estimated prevalence of self-reported depression, a 95% confidence interval, a 5% margin of error, a 20% non-response rate, and a cluster effect of two. The requisite number of patients was 922.

### Data collection tool and procedure

A face-to-face questionnaire administered after a patient interview was used to collect sociodemographic and economic data, behavioral characteristics, level of knowledge about hypertension, clinical characteristics, antihypertensive treatment characteristics, and the relationship between the healthcare system, patient, and doctor. The questionnaire was given in arabic which is the main language spoken by Moroccan.

## Operational definition

### Self-reported depression assessment

The arabic translation version of Patient Health Questionnaire-9 (PHQ-9) was used to assess self-reported depression in hypertensive patients^13^. The PHQ-9 is made up of nine questions to which patients must respond: “never”, “several days”, “more than half the days” and “almost every day”. The following questions were asked: Over the last two weeks: How often do you feel little interest or pleasure in doing things? How often do you feel sad, depressed, or hopeless? How often do you find it difficult to fall or stay asleep, or sleep too much? How often do you feel tired or have low energy? How often do you have a poor perception of yourself—or do you think you are a loser or have not met your own or your family's expectations? How often do you struggle to concentrate on things like reading the newspaper or watching television? How often do you move or speak so slowly that others notice, or are you so restless that you move much more than usual? and How often have you thought you'd be better off dead, or that you'd be better off hurting yourself in some way? Each question was scored on a scale of zero to three. Zero for “never”, one for “several days”, two for “more than half the days” and three for “almost every day”. The sub-scores of the nine questions were put together to yield a total score that ranged from 0 to 27. The following is how the score was interpreted: A score of 0 to 4 indicated no or minimal symptoms; a score of 5 to 9 indicated mild symptoms; a score of 10 to 14 indicated moderate symptoms; a score of 15 to 19 indicated moderately severe symptoms; and a score of 20 to 27 indicated severe depressive symptoms. People who received a score of 5 or higher were deemed to have self-reported depression^13,14^.

### Other variables

An electronic sphygmomanometer with an adjustable cuff (MicroLife Pro M with an accuracy of 3 mmHg) was used to measure blood pressure (BP). Blood pressure control was assessed using the criteria of the European Society of Arterial Hypertension and the European Society of Cardiology (ESH/ESC) recommendations^15,16^.

When patients reported receiving social support, social support was reported. When patients reported measuring their blood pressure outside of the primary health care facilities, self-monitoring was postponed. When patients claimed they had consumed tobacco or alcohol in the previous two months, it was reported. World Health Organization (WHO) criteria were used to determine overweight and obesity^16,17^. When patients experienced physical pain or discomfort, pain and physical discomfort were reported. When patients reported feeling limited in their mobility, restricted mobility was declared. When patients reported feeling stress, stress was declared. Patients reported a lack of autonomy when they felt a lack of autonomy.

### Ethical consideration

Before beginning the survey, we made certain that it complied with the Helsinki Declaration. After informing them about the objective of the study and respecting their privacy and confidentiality, all participants provided written and informed consent. The ethical committee of Rabat's Faculty of Medicine and Pharmacy evaluated and approved the study protocol (#21/21).

### Statistical analysis

All the data was imported into Excel and analyzed using Epi-info 7. Where applicable, continuous variables were presented as the mean and standard deviation. Numbers and percentages were used to express categorical variables. The Pearson chi-square test or, where applicable, Fisher's exact test was used to compare categorical variables. Where appropriate, continuous variables were compared using the ANOVA test or the Mann–Whitney test. The multiple logistic regression included all variables with a *p* value of less than 0.20 in the bivariate analysis. The selection of these variables was made by the manual purposeful selection method. The adjusted odds ratio (AOR) and its 95% confidence interval were used to determine the association between the risk factor and the presence of self-reported depression.

## Results

### Socio-demographic and economic characteristics

Table 1 summarizes the socio-demographic and economic characteristics of hypertensive patients. A total of 1053 patients were approached, with a self-reported depression prevalence of 56.1%. The patients' average age was 63.0 ± 9.9 years; 811 (77.0%) were women; 743 (70.6%) could not read or write; 962 (91.4%) were unemployed; and 728 (69.1%) had a monthly household income of less than $199.

**Table 1:** Socio-demographic and economic characteristics of hypertensive patients followed in primary health care facilities, Marrakech, 2021–2022.

	Total participants n (%)	Self-reported depression n (%)	Absence of self-reported depression n (%)	*p* value
Total participants	1053 (100.0)	591 (56.1)	462 (43.9)	
Means age ± SD (in years)	63.0 ± 09.9	63.2 ± 09.5	62.6 ± 10.4	0.32
Sex				< 0.0001
Female	811 (77.0)	508 (86.0)	303 (65.6)	
Male	242 (23.0)	83 (14.0)	159 (34.4)	
Area of residence				< 0.0001
Urban	788 (74.8)	474 (80.2)	314 (68.0)	
Rural	265 (25.2)	117 (19.8)	148 (32.0)	
Age group (in years)				0.49
≥ 80	58 (05.5)	29 (04.9)	29 (06.3)	
[70–79]	213 (20.2)	123 (20.8)	90 (19.5)	
[60–69]	439 (41.7)	253 (42.8)	186 (40.3)	
[50–59]	248 (23.6)	136 (23.0)	112 (24.2)	
[40–49]	85 (08.1)	47 (08.0)	38 (08.2)	
[29–39]	10 (00.9)	03 (00.5)	07 (01.5)	
Marital status				< 0.0001
Single	379 (36.0)	246 (41.6)	133 (28.8)	
Partnered	674 (64.0)	345 (58.4)	329 (71.2)	
Education				0.03
Can neither read nor write	743 (70.6)	433 (73.3)	310 (67.1)	
Can read and write	310 (29.4)	158 (26.7)	152 (32.9)	
Occupation				0.01
Without	962 (91.4)	551 (93.2)	411 (89.0)	
With	91 (08.6)	40 (06.8)	51 (11.0)	
Monthly income ($)				< 0.0001
< 150	300 (28.5)	211 (35.7)	89 (19.3)	
150–199	428 (40.6)	230 (39.0)	198 (42.9)	
200–299	269 (25.6)	122 (20.6)	147 (31.8)	
300–499	35 (03.3)	20 (03.4)	15 (03.2)	
≥ 500	21 (02.0)	8 (01.3)	13 (02.8)	
Health insurance				0.25
No	168 (16.0)	101 (17.1)	67 (14.5)	
Yes	885 (84.0)	490 (82.9)	395 (85.5)	

SD, standard deviation.

Partnered means married.

Single refers to being single, divorced, or widowed.

### Data on hypertension knowledge and behavioral characteristics

General knowledge on arterial hypertension was unsatisfactory in 1032 (98.0%) patients Table 2. Analysis of behavioral data showed that 284 (27.0%) were on a low-salty diet; 937 (89.0%) had stress; 746 (70.8%) had unsatisfactory physical activity; 669 (63.5%) were sedentary; 259 (24.6%) had difficulty following the low-salt diet; and 367 (34.8%) declared that they did not benefit from social support Table 2.

**Table 2:** Behavioral characteristics of hypertensive patients followed at primary health care facilities, Marrakech, 2021–2022.

	Total participants n (%)	Self-reported depression n (%) n = 591	Absence of self-reported depression n (%) n = 462	*p* value
General knowledge of hypertension				0.42
Unsatisfactory	1032 (98.0)	581 (98.3)	451 (97.6)	
Satisfactory	21 (02.0)	10 (01.7)	11 (02.4)	
Knowledge of the signs of hypertension				0.29
Unsatisfactory	1029 (97.7)	575 (97.3)	454 (98.3)	
Satisfactory	24 (02.3)	16 (02.7)	8 (01.7)	
Knowledge of hypertension complications				0.73
Unsatisfactory	655 (62.2)	365 (61.8)	290 (62.8)	
Satisfactory	398 (37.8)	226 (38.2)	172 (37.2)	
Knowledge of prevention measures against hypertension				0.10
Unsatisfactory	1041 (98.9)	587 (99.3)	454 (98.3)	
Satisfactory	12 (01.1)	4 (00.7)	8 (01.7)	
Tobacco consumption				0.0004
Yes	101 (09.6)	40 (06.8)	61 (13.2)	
No	952 (90.4)	551 (93.2)	401 (86.8)	
Alcohol consumption				0.10
Yes	47 (04.5)	21 (03.5)	26 (05.6)	
No	1006 (95.5)	570 (96.5)	436 (94.4)	
Stress				< 0.0001
Intense	267 (25.4)	221 (37.4)	46 (10.0)	
Moderate	670 (63.6)	343 (58.0)	327 (70.8)	
Low	116 (11.0)	27 (04.6)	89 (19.2)	
Physical activity				< 0.0001
Unsatisfactory	746 (70.8)	449 (76.0)	297 (64.3)	
Satisfactory	307 (29.2)	142 (24.0)	165 (35.7)	
Sedentary				< 0.0001
Yes	669 (63.5)	407 (68.9)	262 (56.7)	
No	384 (36.5)	184 (31.1)	200 (43.3)	
Salt diet				0.0001
Low-salty	284 (27.0)	186 (31.5)	98 (21.2)	
Salty	769 (73.0)	405 (68.5)	364 (78.8)	
Consumption of five fruits and vegetables				0.0001
No	567 (53.8)	349 (59.0)	218 (47.2)	
Yes	486 (46.2)	242 (41.0)	244 (52.8)	
Difficulty following the diet				0.001
Yes	259 (24.6)	167 (28.3)	92 (20.0)	
No	794 (75.4)	424 (71.7)	370 (80.0)	
Social support				< 0.0001
No	367 (34.8)	258 (43.7)	109 (23.6)	
Yes	686 (65.2)	333 (56.3)	353 (76.4)	

### Clinical characteristics of hypertensive patients

Table 3 shows that 508 (48.2%) of hypertensive patients had a comorbidity; 458 (43.5%) had diabetes; 601 (57.1%) had a family history of arterial hypertension; 749 (71.1%) had uncontrolled arterial hypertension; 587 (55.8%) had pain and physical discomfort; and 843 (80.1%) were overweight or obese.

**Table 3:** Clinical characteristics of hypertensive patients followed at primary health care facilities, Marrakech, 2021–2022.

	Total participants n (%) n = 1053	Self-reported depression n (%) n = 591	Absence of self-reported depression n (%) n = 462	*p* value
Comorbidity				< 0.0001
Yes	508 (48.2)	324 (54.8)	184 (39.8)	
No	545 (51.8)	267 (45.2)	278 (60.2)	
Diabetes				< 0.0001
Yes	458 (43.5)	290 (49.1)	168 (36.4)	
No	595 (56.5)	301 (50.9)	294 (63.6)	
Dyslipidemia				0.12
Yes	56 (05.3)	37 (06.3)	19 (04.1)	
No	997 (94.7)	554 (93.7)	443 (95.9)	
Duration of arterial hypertension				0.002
More than 5 years	499 (47.4)	304 (51.4)	195 (42.2)	
Less than or equal to 5 years	554 (52.6)	287 (48.6)	267 (57.8)	
Family history of arterial hypertension				0.0001
Yes	601 (57.1)	368 (62.3)	233 (50.4)	
No	452 (42.9)	223 (37.7)	229 (49.6)	
Controlled blood pressure				0.06
No	749 (71.1)	434 (73.4)	315 (68.2)	
Yes	304 (28.9)	157 (26.6)	147 (31.8)	
Autonomy				0.0005
Yes	79 (07.5)	59 (10.0)	20 (04.3)	
No	974 (92.5)	532 (90.0)	442 (95.7)	
Reduced mobility				< 0.0001
Yes	126 (12.0)	96 (16.2)	30 (06.5)	
No	927 (88.0)	495 (83.8)	432 (93.5)	
Pain and physical discomfort				< 0.0001
Yes	587 (55.8)	406 (68.7)	181 (39.2)	
No	466 (44.2)	185 (31.3)	281 (60.8)	
Overweight/obesity				0.001
Yes	843 (80.1)	494 (83.6)	349 (75.5)	
No	210 (19.9)	97 (16.4)	113 (24.5)	

### Characteristics related to treatment and the patient-doctor-healthcare system triad

Out of 1053 participants, 873 (82.9%) had a bad relationship with the healthcare system, 507 (48.1%) didn't think their doctor was willing to pay attention to their questions about their sickness, and 637 (60.5%) reported antihypertensive drugs non-availability at the primary health care facilities, 263 (25.0%) reported adverse effects of antihypertensive drugs, and 252 (23.9%) reported not buying antihypertensive drugs in case of stockout at the primary health care facilities Table 4.

**Table 4:** Data on antihypertensive treatment and characteristics related to the doctor-patient-care system in hypertensive patients followed at primary health care facilities, Marrakech, 2021–2022.

	Total participants n (%) n = 1053	Self-reported depression n (%) n = 591	Absence of self-reported depression n (%) n = 462	*p* value
Patient-care system relationship				< 0.0001
Unsatisfactory	873 (82.9)	515 (87.1)	358 (77.5)	
Satisfactory	180 (17.1)	76 (12.9)	104 (22.5)	
Patient-doctor relationship				0.01
Unsatisfactory	866 (82.2)	501 (84.8)	365 (79.0)	
Satisfactory	187 (17.8)	90 (15.2)	97 (21.0)	
Feel the doctor's willingness to understand the patient's hypertension concerns				
No	507 (48.1)	321 (54.3)	186 (40.3)	
Yes	546 (51.9)	270 (45.7)	276 (59.7)	
Availability of the drug				< 0.0001
No	637 (60.5)	396 (67.0)	241 (52.2)	
Yes	416 (39.5)	195 (33.0)	221 (47.8)	
Thinking you have a lot of medicine to take				0.03
Yes	40 (03.8)	29 (04.9)	11 (02.4)	
No	1013 (96.2)	562 (95.1)	451 (97.6)	
Adverse drug effects				0.003
Yes	263 (25.0)	168 (28.4)	95 (20.6)	
No	790 (75.0)	423 (71.6)	367 (79.4)	
Non-purchase of a medicine if it is out of stock				< 0.0001
Yes	252 (23.9)	178 (30.1)	74 (16.0)	
No	801 (76.1)	413 (69.9)	388 (84.0)	
Self-monitoring				< 0.0001
No	849 (80.6)	505 (85.5)	344 (74.5)	
Yes	204 (19.4)	86 (14.5)	118 (25.5)	

### Self-reported depression

Self-reported depression was expressed by 591 (56.1%) of the participants. They were 63.2 ± 9.5 years old on average, 508 (86.0%) were women, and 474 (80.2%) lived in cities Table 1. According to an analysis of behavioral and clinical data, 564 (53.6%) were stressed, 186 (31.5%) were on a reduced salt diet Table 2, 195 (42.2%) had a family history of arterial hypertension, and 406 (68.7%) were in pain or physical discomfort Table 3. In 515 (87.1%) participants, the interaction with the healthcare system was unsatisfactory Table 4.

According to the distribution of hypertensive patients by level of self-reported depression, 429 (40.7%) had mild self-reported depression, 130 (12.3%) had moderate self-reported depression, 29 (2.8%) had moderately severe self-reported depression, and 3 (0.3%) had severe self-reported depression, while 462 (43.9%) had no or minimal self-reported depression.

During the bivariate analysis, the *p* value was set at 0.20. As mentioned in Table 5, after bivariate analysis, these factors were associated with the presence of self-reported depression: female sex (*p* < 0.0001); being single compared to partnered (*p* < 0.0001); cannot read or write (*p* = 0.03); urban compared to rural (*p* < 0.0001); being unemployed (*p* = 0.01); low monthly income (*p* < 0.0001); high stress compared to low stress (*p* < 0.0001); moderate stress compared to low stress (*p* < 0.0001); unsatisfactory knowledge about preventive measures against HBP (*p* = 0.11); tobacco consumption (*p* = 0.0005); alcohol consumption (*p* = 0.10); unsatisfactory physical activity (*p* < 0.0001); sedentary (*p* = 0.0001); low-salt diet (*p* = 0.0002); non-consumption of five fruits and vegetables per day (*p* = 0.0001); lack of self-monitoring of blood pressure (*p* < 0.0001); lack of social support (*p* < 0.0001); presence of comorbidity (*p* < 0.0001); presence of diabetes (*p* < 0.0001); presence of dyslipidemia (*p* = 0.11); duration of hypertension greater than five years (*p* = 0.002); family history of arterial hypertension (*p* = 0.0001); uncontrolled blood pressure (*p* = 0.06); lack of autonomy (*p* = 0.0004); pain and physical discomfort (*p* < 0.0001); reduced mobility (*p* < 0.0001); overweight and obesity (*p* = 0.001); difficulty following a low-salty diet (*p* = 0.001); unsatisfactory relationship between the patient and the healthcare system (*p* < 0.0001); unsatisfactory doctor-patient relationship (*p* = 0.001); patients' perception of their doctor's lack of willingness to understand their concerns regarding their HBP (*p* < 0.0001); stock-out of antihypertensive drug at primary health care facilities (*p* < 0.0001); non-purchase by the patient of the antihypertensive drug in the event of a stock-out at the primary health care facilities (*p* < 0.0001); perception by the patient of having too much medicine to take (*p* = 0.03); perception that the drug has more adverse effects than benefits (*p* = 0.003) Table 5.

**Table 5:** Multivariate analysis (odds ratio, *p* value) of risk factors associated with self-reported depression among Hypertensive patients, Marrakech, Morocco, 2021–2022.

	Bivariate analysis		Multivariate analysis complete model	
	COR (95% CI)	*p* value	AOR (95% CI)	*p* value
Female sex	3.2 [2.37–4.34]	< 0.0001	4.1 [2.45–6.88]	< 0.0001
Age in years	1.0 [0.99–1.01]	0.32	1.02 [1.00–1.04]	0.05
Being single	1.7 [1.36–2.28]	< 0.0001	0.9 [0.67–1.38]	0.85
Can neither read nor write	1.3 [1.02–1.75]	0.03	0.9 [0.64–1.31]	0.63
Unemployed	1.7 [1.10–2.63]	0.01	0.89 [0.49–1.61]	0.72
Urban	1.9 [1.44–2.52]	< 0.0001	2.2 [1.47–3.44]	0.0002
Monthly income ($)		< 0.0001		
≤ 149/ ≥ 500	3.8 [1.54–9.61]	0.003	1.2 [0.38–4.16]	0.70
150–199/ ≥ 500	1.8 [0.76–4.64]	0.16	0.9 [0.28–2.96]	0.88
200–299/ ≥ 500	1.3 [0.54–3.36]	0.52	0.6 [0.20–2.11]	0.48
300–499/ ≥ 500	2.1 [0.71–6.55]	0.17	0.8 [0.21–3.41]	0.82
Unsatisfactory knowledge of prevention measures against hypertension	2.58 [0.77–8.64]	0.11	0.4 [0.09–1.86]	0.25
Tobacco consumption	0.40 [0.31–0.72]	0.0005	1.3 [0.59–2.73]	0.54
Alcohol consumption	0.61 [0.34–1.11]	0.10	1.1 [0.45–2.74]	0.81
Intense stress/low stress	15.8 [9.27–27.0]	< 0.0001	10.5 [5.63–19.75]	< 0.0001
Moderate stress/low stress	3.4 [2.19–5.45]	< 0.0001	2.2 [1.32–3.85]	0.002
Unsatisfactory physical activity	1.7 [1.34–2.29]	< 0.0001	1.1 [0.74–1.57]	0.66
Sedentarity	1.7 [1.31–2.17]	0.0001	1.0 [0.71–1.52]	0.81
Low-salt diet	1.7 [1.28–2.26]	0.0002	3.1 [1.28–7.37]	0.01
Not consuming five fruits and vegetables a day	1.6 [1.26–2.06]	0.0001	1.3 [0.96–1.80]	0.07
Lack of monitoring hypertension	2.0 [1.47–2.74]	< 0.0001	1.9 [1.27–2.81]	0.001
Lack of social support	2.5 [1.91–3.28]	< 0.0001	1.3 [0.90–1.82]	0.16
Presence of comorbidity	1.8 [1.43–2.34]	< 0.0001	2.5 [0.95–6.79]	0.06
Presence of diabetes	1.7 [1.31–2.16]	< 0.0001	0.6 [0.25–1.70]	0.38
Presence of dyslipidemia	1.5 [0.88–2.74]	0.11	0.5 [0.21–1.30]	0.16
Duration of high blood pressure more than five years	1.4 [1.13–1.85]	0.002	1.0 [0.74–1.38]	0.91
Family history of arterial pressure	1.6 [1.26–2.07]	0.0001	1.6 [1.16–2.22]	0.003
Uncontrolled blood pressure	1.2 [0.98–1.68]	0.06	1.02 [0.73–1.45]	0.86
Lack of autonomy	2.4 [1.45–4.13]	0.0004	1.2 [0.54–2.86]	0.59
Pain and physical discomfort	3.4 [2.63–4.39]	< 0.0001	2.9 [2.08–3.99]	< 0.0001
Reduced mobility	2.7 [1.81–4.29]	< 0.0001	1.5 [0.73–2.91]	0.28
Overweigh and obesity	1.6 [1.21–2.23]	0.001	1.1 [0.71–1.63]	0.70
Difficulty following the low-salt diet	1.5 [1.18–2.11]	0.001	0.5 [0.19–1.21]	0.12
Unsatisfactory patient relationship with the healthcare system	1.9 [1.42–2.72]	< 0.0001	1.8 [1.10–2.91]	0.02
Unsatisfactory doctor-patient relationship	1.4 [1.07–2.03]	0.01	0.0 [0.55–1.37]	0.56
Feel the doctor's willingness to understand the patient's hypertension concerns	1.7 [1.37–2.25]	< 0.0001	1.1 [0.72–1.55]	0.74
Availability of the drug	1.8 [1.44–2.39]	< 0.0001	0.7 [0.48–1.09]	0.12
Non-purchase of a medicine if it is out of stock	2.2 [1.66–3.06]	< 0.0001	1.3 [0.90–2.02]	0.14
Thinking you have a lot of medicine to take	2.1 [1.04–4.27]	0.03	1.1 [0.46–2.52]	0.85
Adverse drug effects	1.5 [1.15–2.04]	0.003	1.5 [1.04–2.21]	0.03

*COR* crude odds ratio, *AOR* adjusted odds ratio, *CI* Confidence interval.

### Multivariate analysis

After adjusting for the other variables, we identified the following factors as being associated with self-reported depression in hypertensive patients: high stress compared to low stress (Adjusted Odd Ratio of 10.5; 95% CI [5.63–19.75]); female sex (AOR of 4.1; 95% CI [2.45–6.88]); the low-salt diet (AOR of 3.1; 95% CI [1.28–7.37]); pain and physical discomfort (AOR of 2.9; 95% CI [2.08–3.99]); urban (AOR of 2.2; 95% CI [1.47–3.44]); moderate stress compared to low stress (AOR of 2.2; 95% CI [1.32–3.85]); lack of self-monitoring of arterial hypertension (AOR of 1.9; 95% CI [1.27–2.81]); unsatisfactory patient relationship with the healthcare system (AOR of 1.8; 95% CI [1.10–2.91]); family history of arterial hypertension (AOR of 1.6; 95% CI [1.16–2.22]); and perception that the drug has more adverse effects than benefits (AOR of 1.5; 95% CI [1.04–2.21]) Table 5.

## Discussion

With a prevalence of 56.1%, the current study found a significant prevalence of self-reported depression. The frequency of self-reported depression ranged from 26.6 to 40.1% in countries with similar socio-demographic and economic characteristics to Morocco. Indeed, self-reported depression was 26.6% in a sample of 237 hypertensive patients in Nigeria in 2019^18^. In Peru, the prevalence of self-reported depression was 34.9% among 10,566 participants in 2017^19^. In Pakistan, the prevalence was 40.1% among 411 hypertensive patients in 2017^20^. The prevalence of self-reported depression in hypertensive patients ranged from 37 to 58% in low-income nations. Between 2015 and 2016, 58.1% of 234 hypertensive patients in Andkhoy, Afghanistan, self-reported depression^21^. In Ethiopia, between 2019 and 2020, 37.8% of 407 hypertensive patients reported having depression^22^.

A meta-analysis of 41 studies conducted in high-income countries, such as China, revealed that self-reported depression was present in 28.5% of hypertensive patients^23^. Our study's high prevalence of self-reported depression could be explained by differences in environmental variables, genetics, or even sample size.

Female sex was associated with the presence of self-reported depression among hypertensives in our study. This identical finding has been reported in the literature^20,22,24^. This could be explained by hormonal variations between the sexes as well as psychosocial variables connected with feminine gender conceptions^25^. Indeed, parents engage with their child differently depending on whether it is a boy or a girl at birth. This affiliation with the feminine or masculine gender influences how adults perceive their own depression symptoms. Thus, because the masculine gender is associated with attributes such as emotional control and independence, men are much less likely to seek medical attention when they have symptoms of depression, whereas women, whose gender identity construction makes them more fragile than men, are more likely to consult when they are not feeling well.

Stress was associated with self-reported depression in our study. Patients' dissatisfaction with the healthcare system could explain this stress^26^, concerns of the problems that their disease may entail, and their low socioeconomic status, which is an impediment to proper hypertension control.

Salt is required for the maintenance of osmotic pressure, plasma and interstitial volumes, acid–base balance, cell electrical activity, and the cardiovascular system's reaction to endogenous vasopressors. To reduce blood pressure, the WHO advises a salt diet of fewer than 5 g per person^27^. Subjects with hypertension are subjected to a low-salt diet, which might cause the emergence of depressive symptoms, especially since the low-salty diet is a lifelong diet. This self-reported depression could be attributed primarily to the blandness of meals and patients' perceptions of being in control. In our study, being on a low-salty diet was associated with self-reported depression.

In our study, pain and physical discomfort in hypertensives were related to the prevalence of depression. This could occur as a result of feelings of worthlessness and powerlessness, as well as low self-esteem. According to the scientific literature, pain and depression have common processes. They have norepinephrine and serotonin neurotransmission. In depression, a decrease in these neurotransmitters may disrupt the functioning of inhibitory corticospinal pathways that modulate the activity of nociceptive neurons in the posterior horn, causing the patient to experience pain stimuli relevant to his organism's normal functioning^28^. Depression is also associated with cognitive and affective alterations, which can lead to an especially negative perception of body experiences. This is followed by a chain reaction of hypervigilance toward symptoms and avoidance of movement, which can lead to an amplification of pain perception^29^.

The presence of self-reported depression associated with urban areas in our study Indeed, living in an urban setting is a risk factor for mental health decline^30^. Factors such as a lack of green space and bodies of water, population density, and the frantic pace of urban life can all contribute to increased sensitivity to social stress and mental exhaustion^31^. In Europe and the United States, depression is 39% more common in urban areas than in rural areas^32^.

Self-monitoring of blood pressure enables people with hypertension to be empowered and regulate their blood pressure. Lack of self-monitoring was associated with self-reported depression in our study. This could be explained by patients' lack of motivation and denial of the disease, their inadequate awareness of arterial hypertension, their poor level of education, and the complexity of blood pressure measurement. Add to it a lack of social support from family, friends, and entourage.

In our study, an unsatisfactory relationship with the healthcare system was associated with self-reported depression. This would be related to the helplessness that hypertensive patients feel as a result of the lack of anti-hypertensive medicines at the primary health care facilities, which can last many months, the high cost of these drugs, and the low monthly income per household. This encourages consumers not to purchase medicine in the case of a stock shortage and leaves them concerned about their health as a result of the consequences that HBP might create if blood pressure levels are not controlled.

In our study, having a family history of arterial hypertension was associated with self-reported depression. This could be due to concerns about potential consequences such as strokes and myocardial infarction, as well as patients' disinterest in illness care.

In our work, the presence of self-reported depression was connected with the patient's assessment of the detrimental effects of antihypertensive medicines. This could be explained by the patient's lack of drive and anxiety about the disease's complications.

## Limitations of the study

Our study was able to meet its objectives; however, certain limitations were present, such as the bias of prevarication encountered during the collection of economic and behavioral data and the cross-sectional design of the study, which makes it impossible to demonstrate a causal relationship between exposure and the occurrence of self-reported depression.

Although the study suggests a high prevalence of depression, possibly linked to the COVID-19 infection, this is not entirely accurate. Indeed, the period between 2021 and 2022 was characterized by a return to normal life.

## Conclusion

Hypertensive patients are vulnerable when it comes to mental health issues. Our findings show an important prevalence of self-reported depression. Following these findings, it is suggested that mental health be included in the care and follow-up of hypertensive patients.

## Data Availability

All data generated or analyzed during this study are included in this published article.
